# Uncertainties surrounding the oldest fossil record of diatoms

**DOI:** 10.1038/s41598-023-35078-8

**Published:** 2023-05-17

**Authors:** Karolina Bryłka, Andrew J. Alverson, Rebecca A. Pickering, Sylvain Richoz, Daniel J. Conley

**Affiliations:** 1 Department of Geology, Lund University, Sölvegatan 12, 223 62 Lund, Sweden; 2 Department of Biological Sciences, University of Arkansas, Fayetteville, AR 72701 USA

**Keywords:** Molecular evolution, Palaeontology, Taxonomy

## Abstract

Molecular clocks estimate that diatom microalgae, one of Earth’s foremost primary producers, originated near the Triassic–Jurassic boundary (200 Ma), which is close in age to the earliest, generally accepted diatom fossils of the genus *Pyxidicula*. During an extensive search for Jurassic diatoms from twenty-five sites worldwide, three sites yielded microfossils initially recognized as diatoms. After applying stringent safeguards and evaluation criteria, however, the fossils found at each of the three sites were rejected as new diatom records. This led us to systematically reexamine published evidence in support of Lower- and Middle-Jurassic *Pyxidicula* fossils*.* Although *Pyxidicula* resembles some extant radial centric diatoms and has character states that may have been similar to those of ancestral diatoms, we describe numerous sources of uncertainty regarding the reliability of these records. We conclude that the Lower Jurassic *Pyxidicula* fossils were most likely calcareous nannofossils, whereas the Middle Jurassic *Pyxidicula* species has been reassigned to the Lower Cretaceous and is likely a testate amoeba, not a diatom. Excluding the *Pyxidicula* fossils widens the gap between the estimated time of origin and the oldest abundant fossil diatom record to 75 million years. This study underscores the difficulties in discovering and validating ancient microfossils.

## Introduction

Our knowledge of past biodiversity and evolution is shaped largely by the fossil record, which suffers from variable levels of incompleteness^1^. This incompleteness alters our understanding of diversification dynamics^2^, rates of evolution^3^, and possible impacts of different lineages on the paleoenvironment. In the absence of a complete fossil record, molecular clocks can provide important insights into the pattern and timing of key events in the evolutionary history of a lineage. Although powerful, divergence times inferred from molecular clocks are most accurate when they include a broad set of fossil calibration points, relax the assumption of a strict molecular clock (i.e., constant rate of evolutionary change), and account for heterogeneity in the rates of speciation and extinction across the phylogeny^4^. The probability of discovering the oldest fossil representative of any lineage is vanishingly small, resulting in gaps between the oldest fossil recorded and ages estimated from molecular clocks. Numerous lineages such as foraminifera and angiosperms have large differences between their estimated clade ages and the oldest discovered fossils^5–7^. Although molecular clocks provide insights into the timeline of diversification, fossils provide direct evidence of the species richness and character states present at a particular time in the history of a lineage.

Diatoms are globally distributed photosynthetic eukaryotes with strong influences on the global biogeochemical cycling of nitrogen, phosphorus, carbon, oxygen, and silica^8^. Although our understanding of the phylogenetic relationships and genome evolution in diatoms has progressed rapidly over the past decade, advances in our understanding of the early fossil record of diatoms have not kept pace. Molecular clocks suggest that diatoms originated near the Triassic–Jurassic boundary (ca. 200 Ma)^5^, which is relatively close to the oldest known diatom fossils from the Lower Jurassic period (ca. 182 Ma). These fossils consist of two diatom species in the genus *Pyxidicula,* extracted from a fossilized sponge (*Phymatoderma*) collected from the Upper Liasic shales in Boll, Germany^9^. A second *Pyxidicula* record of Middle Jurassic age (174–163 Ma) includes one diatom species that was also extracted from a fossilized sponge (*Spongelites fellenbergi*) collected in Bernese Oberland, Switzerland^10^. The next available records are from the upper Lower Cretaceous (Aptian, 125–115 Ma) deposits in eastern Australia^11–14^ and the Weddell Sea deep-sea sediments (113–110 Ma)^15,16^, which boasts a diverse community of diatoms. From this point forward in geological history, the fossil record indicates that diatoms became widespread and abundant on a global scale^17,18^.

To fill the large (ca. 40 Myr) gap between the Middle Jurassic and Lower Cretaceous diatom records, we carried out an extensive search for Mesozoic diatoms. Our search revealed many difficulties connected with fossil record validation, including morphological identification, environmental contamination, and age control issues. The absence of diatoms in Jurassic and Cretaceous sediments and the scarce documentation of known Jurassic diatom fossils led us to re-evaluate the evidence for the earliest diatom fossils, which we ultimately reject. Our study underscores the importance of applying numerous verification safeguards to prevent misidentification of microfossils.

## Results

We sampled and examined 25 sections for Mesozoic diatoms. The sections were from outcrops or cores in France, Germany, Switzerland, Austria, Russia, and Oman, and from ocean drilling in the Atlantic and Pacific Oceans. The oldest age across these sections was Sinemurian (199–190 Ma) and the youngest was Cenomanian (100–94 Ma). A total of 22 of the study sites were barren in diatom microfossils, and three sites contained fossils initially identified as diatoms. Findings from these study sites are highlighted below.

### Rejection of diatom-like fossils by elemental analysis

**Figure 1:**
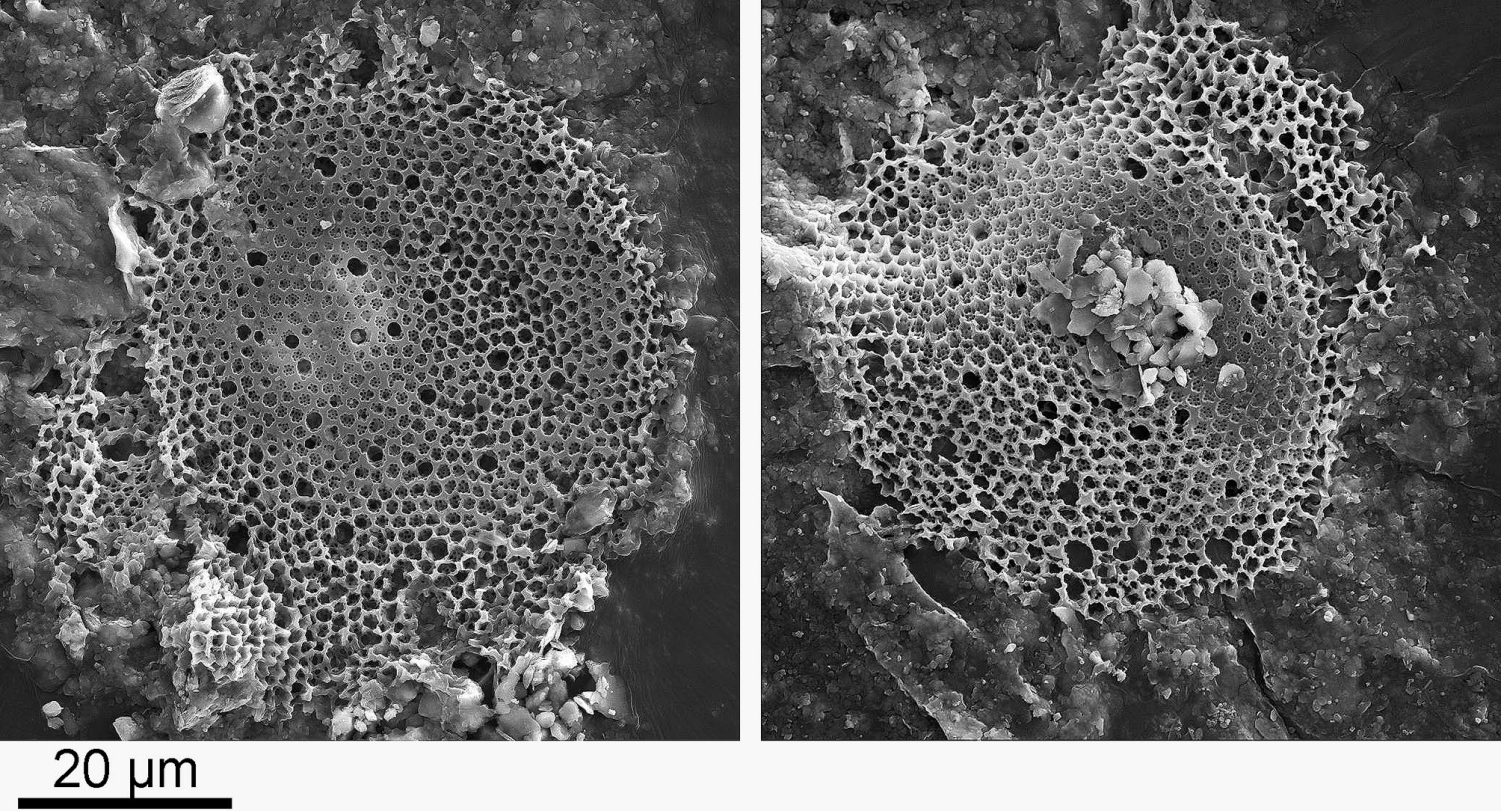
SEM images of *Pleurozonaria* specimens from the Schandelah core. Sample Depth: 41 m below ground level^20^.

**Figure 2:**
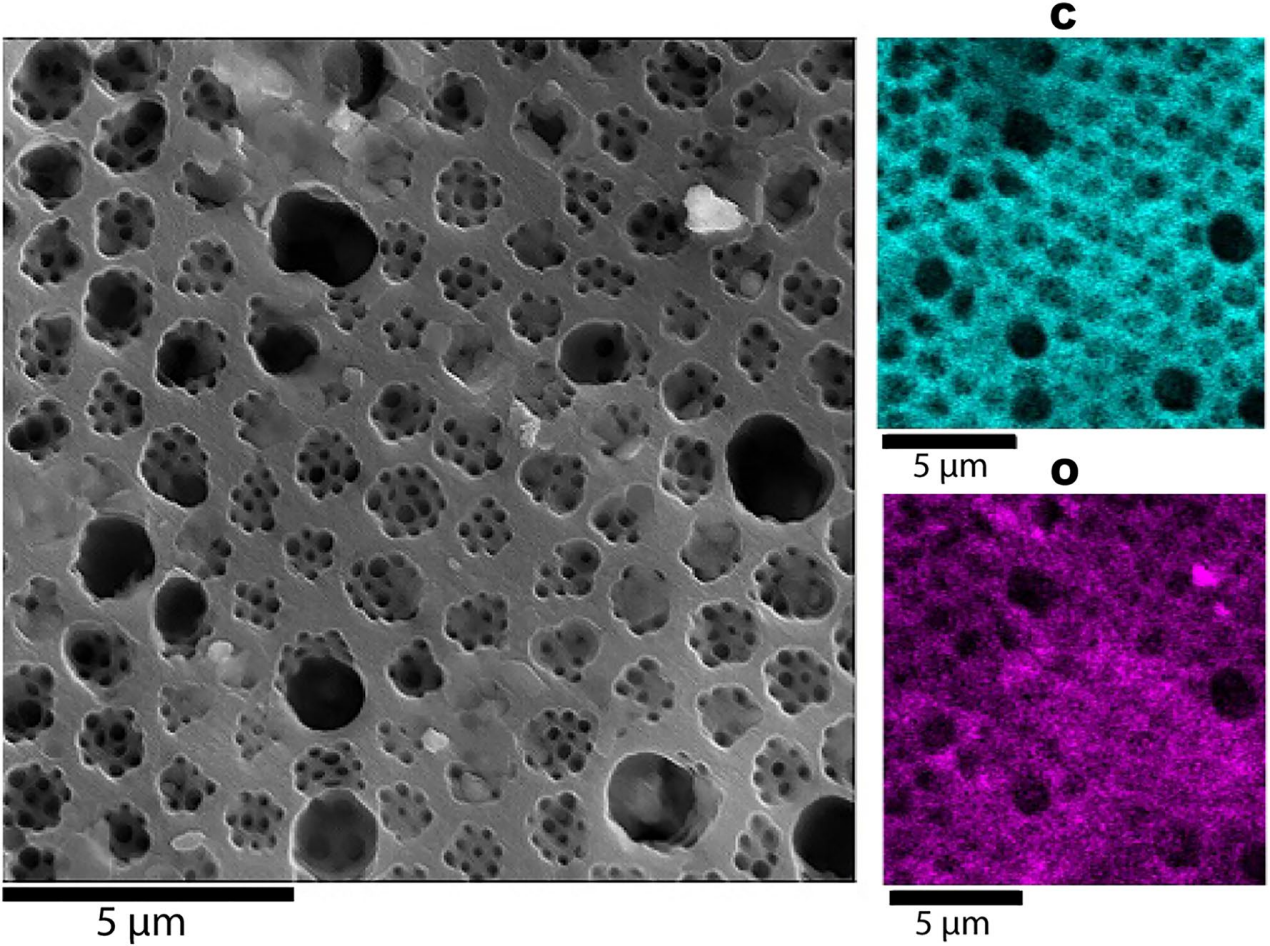
Elemental mapping of oxygen [O] and carbon [C] on a *Pleurozonaria* specimen from the Schandelah core. Sample Depth: 41 m below ground level.

We examined Lower Toarcian (183–181 Ma) black shales (also known as Toarcian Black Shales and Posidonia Shale^19^), from Schandelah, northern Germany. These black shales are formerly anoxic sediments^20^ with a high potential for fossil preservation^21^. Processed material yielded microfossils with morphological characteristics similar to many diatoms, including a circular shape and large reticulated and regularly arranged pores that resemble the cribra^22^ of diatoms (Figs. 1, 2).

The cell wall of diatoms is composed of hydrated silica. Scanning electron microscopy with energy dispersive X-ray spectrometry (SEM–EDS) was used to determine the elemental composition of the microfossils and showed that carbon, not silica, was the main structural component (Fig. 2), indicating that the Toarcian microfossils were not diatoms. Instead, these fossils were reproducing cysts of *Pleurozonaria*, a member of the prasinophyte lineage of green algae^23,24^. *Pleurozonaria* was identified based on carbon-based cell wall and presence of surface pores with secondary structure and canals (hollow pores) (B. van de Schootbrugge pers. comm.). These cysts are abundant across Toarcian (183–174 Ma) shales in the Northwestern European Basin^25^ and can withstand robust chemical cleaning treatments.

### Environmental contamination

Callovian (Middle Jurassic, 166–163 Ma) marls from Ravine Chénier near La Voulte-sur-Rhône, France, are reported as containing siliceous sponge spicules^26^, so we considered them promising for preservation of other siliceous microfossils such as diatoms. Samples yielded a large number of diatom microfossils belonging to the raphid diatom clade, mainly extant species of *Hantzschia*, *Pinnularia,* and *Luticola*. Raphid diatoms did not appear in the fossil record until the Late Cretaceous (ca. 80 Ma)^27^, and calibrated molecular clocks estimated their origin at 120 Ma^5^. Therefore, it was unlikely to observe well-preserved raphid diatoms with clear extant analogs in archives as old as Callovian (166–163 Ma). To test whether these were contaminants from the environment, we removed the outer soft layer of the samples (see Methods) and determined that these taxa were only present in the outermost layer. The inner layers were devoid of fossil diatoms, suggesting that the raphid pennate diatom specimens observed in these samples were the result of surface contamination.

### Age control and the determination of in situ fossil status

**Figure 3:**
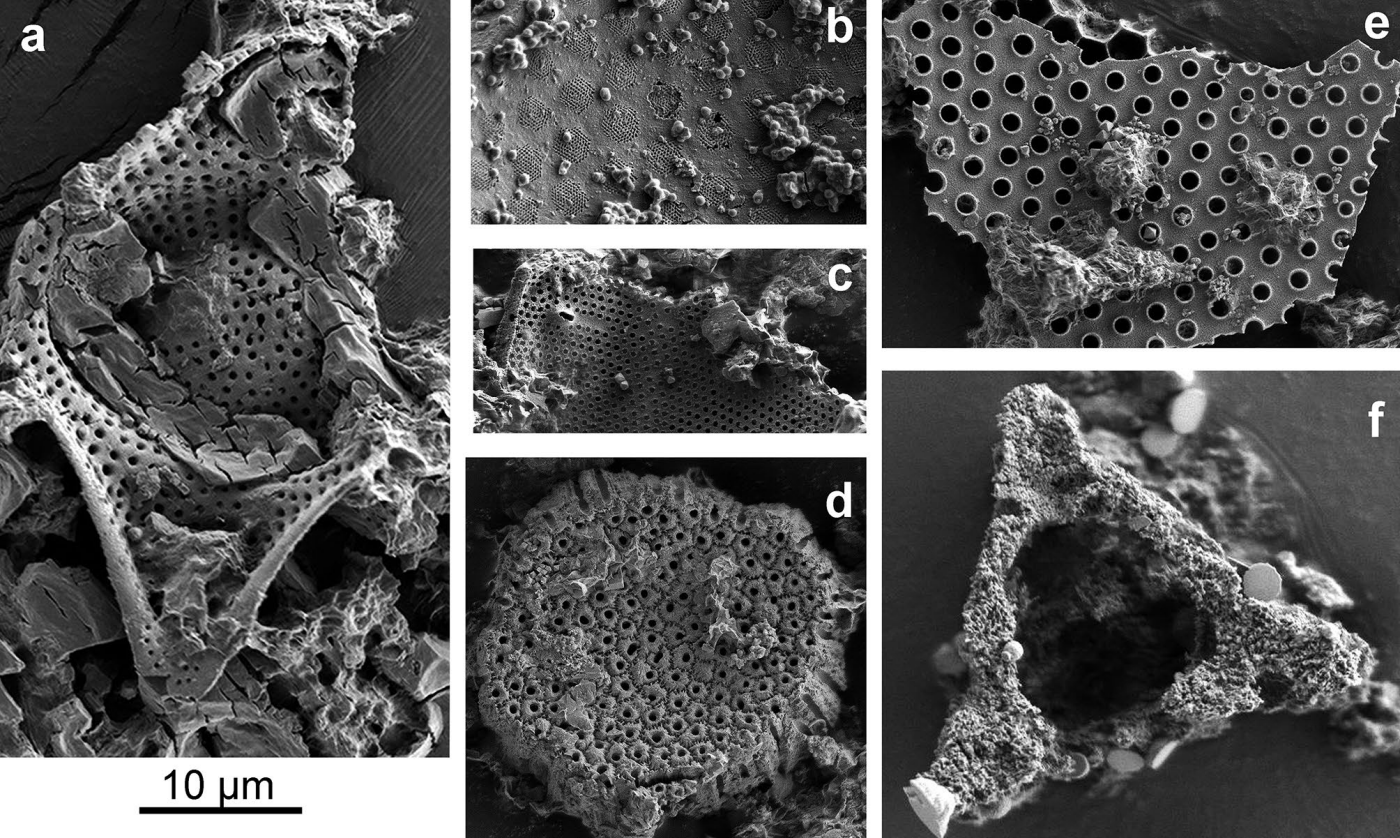
SEM images of diatoms recovered from cherts of DSDP site 416A^28^. Specimens (**a**–**d**) from the sample: 50-416A-54-1; 106–107 cm; specimen (**f**) 416-39R-1W, 100–101 cm.

We examined cherts of Upper Jurassic and Lower Cretaceous age from Deep Sea Drilling Project (DSDP) site 416A in the Central Atlantic Ocean^28^. Cherts were reported to preserve siliceous microfossils such as radiolarians^29^ and diatoms^30^ and therefore were considered promising for the purposes of finding new diatom fossils. Diverse diatom assemblages were extracted from both Upper Jurassic and Lower Cretaceous cherts (see Methods) (Fig. 3 and Supplementary Fig. 1). Some of the frustules appeared black under transmitted light and by EDS mapping were confirmed pyritised (Supplementary Fig. 2).

Other microfossils such as radiolarians, sponge spicules, and coccolithophores were also observed within the 416A chert samples. The DSDP 416A Initial Report site description and drilling information collected during the expedition suggested possible downcore sediment movement during drilling and recovery^28^. Coccolithophores, which are routinely used in the stratigraphy of Mesozoic and Cenozoic sediments^31^, were used to evaluate the age of our samples. The 416A chert samples contained coccolithophores from both Mesozoic (e.g., *Watznaueria* and *Manivitella*) and Cenozoic (e.g., *Discoaster* and *Toweius*) Eras indicating probable mixing of the microfossil assemblages and thus preventing accurate age assignment to these samples. Diatoms present in the cherts include genera that span Mesozoic and Cenozoic eras, but no taxa known solely from the Mesozoic were observed. The presence of Cenozoic coccolithophores, together with the lack of Cretaceous diatoms in the chert, supports sediment movement during the core recovery as suggested in the Initial Reports^28^.

## Validation of Jurassic diatom fossils

**Figure 4:**
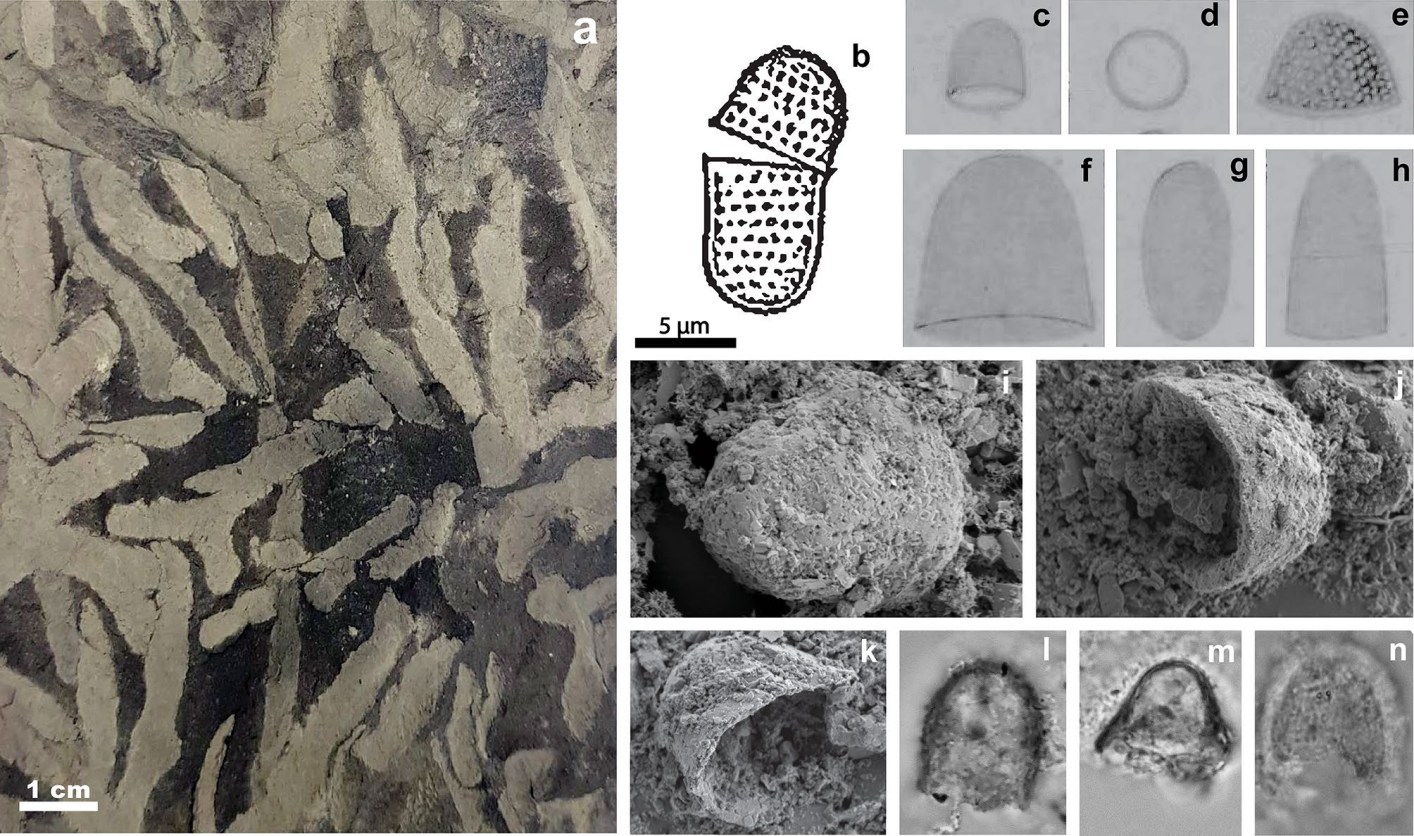
Compilation of specimens used to validate lower Jurassic fossils. (**a**) *Phymatoderma* sample, (**b**) *P. bollensis* complete specimen, (**c**) and (**e**) *P. bollensis* “isolated’’ halves, (**d**) *P. bollensis* cross-section, (**f**) and (**h**) *P. liasica* “isolated’’ halves, (**g**) *P. liasica* “from the narrow side’’ possibly the top view, (**h**) *P. liasica* unspecified view, (**i**) *Schizosphaerella* larger halve, (**j**) *Schozospaerella* smaller halve with a circular cross-section, (**k**) *Schizosphaerella* smaller halve with elliptical cross-section, (**l**–**n**) *Schizosphaerella* halves. Image type: (**a**) Photograph (this study), (**b**–**h**) original Rothpletz’s drawings^9^, (**i**–**k**) SEM images (this study), (**l**–**n**) LM photographs (this study).

**Figure 5:**
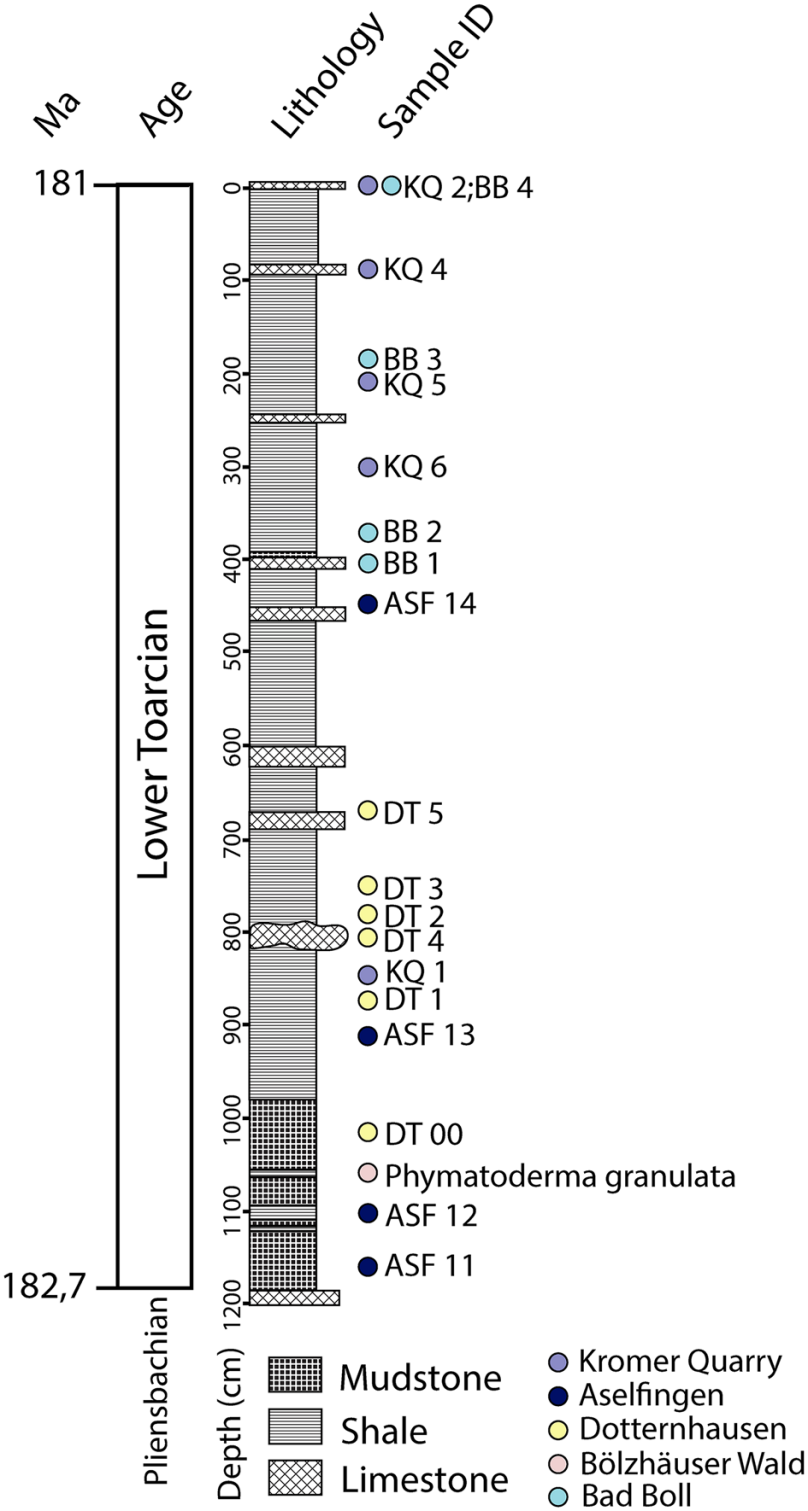
Posidonia Shale profile (redrawn from Röhl et al.^37^) with the position of collected samples.

*Pyxidicula bollensis* Rothpletz (Fig. 4b–e) and *Pyxidicula liasica* Rothpletz (Fig. 4f–h) are the two oldest reported diatom fossils. These species are known from one locality, Boll in Southern Germany, where they were originally described by Rothpletz in 1896^9^. The microscope slides containing *P. bollensis* and *P. liasica* went missing and are unavailable for further study^32^. According to the original publication, the Lower Jurassic *Pyxidicula* specimens were extracted from a layer abundant in fossil sponges belonging to the Upper Liasic shale succession^9^ (“*Ueber Phymatoderma, ein Diatomeen einschliessender Hornshwamm*”*;* Eng. “*About Phymatoderma, a diatom enclosing horn sponge*”)^9^. Upper Liasic shales are also known as Toarcian Black Shales or Posidonia Shales^19^. An extensive search for outcrops around Boll led to one location where only the upper part of the Posidonia Shale profile was exposed (Fig. 5, “BB”). The *Phymatoderma* layer, where the original *Pyxidicula* reportedly originated from, belongs to the lower part of the Posidonia Shale profile (Fig. 5) and is no longer exposed in present-day Boll. Dr. Guenter Schweigert (pers. comm.) from the State Museum of Natural History in Stuttgart (Naturkunde Museum Stuttgart) confirmed that the *Phymatoderma* layer is currently not exposed in known locations and provided a piece of the *Phymatoderma* layer that was collected in 2009 from the Bölzhäuser Wald, a forest 1.5 km north of the village of Ohmden (approximate coordinates 48.662211°, 9.523260°). *Phymatoderma* is comprised of dichotomous branches of light gray/beige color that are embedded in the black shale matrix (Fig. 4a). Importantly, *Phymatoderma* is no longer recognized as a sponge, but instead as a trace fossil representing fecal pellets (currently called *Phymatoderma granulata*)^33^. In addition, we did not find sponges in the Boll outcrop, and no sponge specimens have been reported from Upper Liasic shales^19,34,35^.

The *Phymatoderma* layer was examined in two ways: (1) unprocessed, and (2) following chemical digestion in hydrochloric acid (see Methods). The unprocessed sample contained few fossil foraminifera, rare sponge spicules, and abundant coccolithophores. In addition, the sample included abundant microfossils with morphological characteristics similar to Rothpletz’s *Pyxidicula*: cells of 6–14 μm diameter, flat surface with punctate ornamentation, and a slightly deflected margin in some specimens (Fig. 4i–n). Elemental mapping revealed that these fossils were composed of calcite, magnesium and carbon (Supplementary Fig. 3). Based on their morphological characteristics and chemical composition, we classified these as calcareous nannofossils recognized as *incertae sedis Schizosphaerella* Deflandre and Dangeard. A complete *Schizosphaerella* specimen consists of two halves of different sizes^36^, similar to diatoms and identical to what was documented for the Lower Jurassic *Pyxidicula* described by Rothpletz (Fig. 4b).

The high degree of morphological similarity between the *Pyxidicula* species described by Rothpletz and the *Schizosphaerella* specimens in our samples suggests that Rothpletz’s *Pyxidicula* species were the same calcareous nannofossils present in our samples. Our *Schizosphaerella* specimens completely dissolved in 1% hydrochloric acid (HCl) after 30 min, whereas Rothpletz claimed that his specimens withstood HCl chemical digestion and therefore must be siliceous diatoms^9^. Rothpletz’s chemical treatment procedure was as follows: “*To study their outer form, it is best to dissolve a piece of the fossil sponge in dilute hydrochloric acid. The silica shells then remain, while the coccoliths, sponge needles and foraminifera shells all go into solution. The small shells then float around under the cover glass and can be viewed and measured from all sides as they rotate.’’* (*Italics:* direct translation from German page 910)^9^. This description suggests that the sample was dissolved on a microscope slide, a technique the author also described in a later publication^10^. Additionally, the 1896^9^ text describes how foraminifera, coccolithophores, and calcareous sponge spicules dissolve while in solution, and *Pyxidicula* remains. Rothpletz stated, however, that the surface of *Pyxidicula* also dissolved, losing its pre-treatment cell wall pattern^9^ (see Fig. 4e pre-treatment vs. Fig. 5c post-treatment). If our interpretation is correct, then the dissolution procedure took place directly on a microscopic slide, and the amount and/or concentration of HCl used may have been too weak to completely dissolve the calcareous parts of the sample. Rare calcareous spicules and foraminifera would have dissolved, whereas the abundant thick-walled *Schizosphaerella* remained, albeit slightly dissolved, leading Rothpletz to incorrectly infer that the specimens he observed were siliceous in composition.

In conclusion, the specimens we observed were extracted from the same sediment type (*Phymatoderma*) as Rothpletz’s *Pyxidicula*, the morphological characteristics of both fossils are similar, and both exhibited some degree of dissolution in HCl. The silicon frustules of diatoms can withstand boiling in concentrated HCl without dissolution. This leads us to the conclusion that the two *Pyxidicula* species described by Rothpletz in 1896—*P. bollensis and P. liasica*—were most likely the calcareous nannofossils of the genus *Schizosphaerella*. In addition, the two *Pyxidicula* species described by Rothpletz differed in the shape of their cross-sectional view (circular in *P. bollensis* vs. elliptical in *P. liasica* [Fig. 4e,g])^9^. The *Schizosphaerella* specimens we observed exhibited both shapes (Fig. 4j circular vs. Fig. 4k elliptical), suggesting that the elliptical shape of the fossils observed by both Rothpletz and us is an outcome of fossil compaction and not unique species-specific morphologies, leading us to hypothesize that *P. bollensis* and *P. liasica* are conspecific.

To further test whether diatoms were present in Southern Germany’s Toarcian Black Shales and to avoid bias which might have been caused by examining *Phymatoderma* from a different locality than Boll, we sampled several South German Posidonia Shale successions. A total of 19 samples of Lower Jurassic Black Shales were examined (Fig. 5), and diatoms were not observed in any of them.

**Figure 6:**
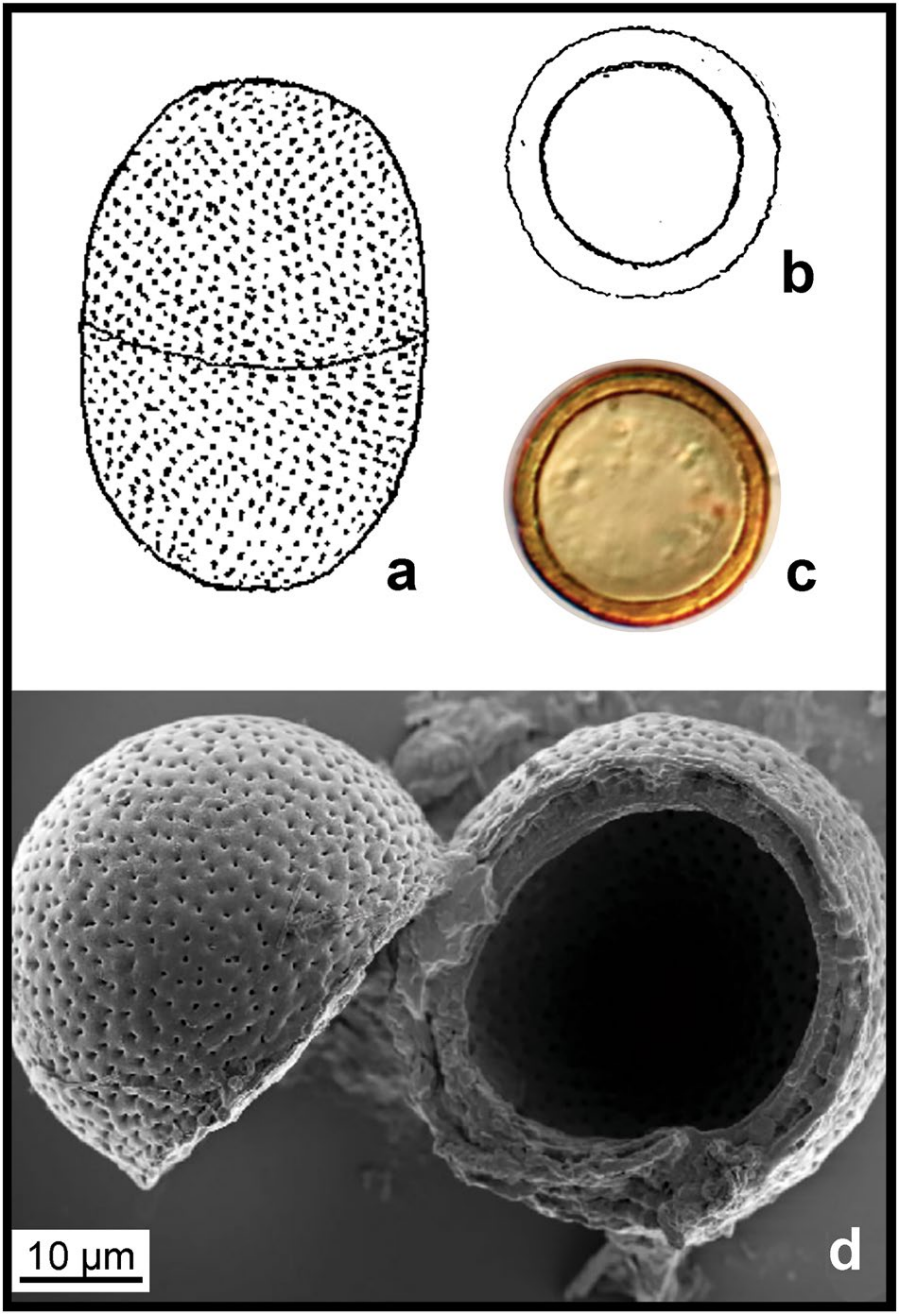
Compilation of specimens used to identify Lower Cretaceous fossils. (**a**, **b**) *P. annulata* Rothpletz 1900, (**c**) *P. operculata* Ehrenberg 1838, (**d**) *P. patens* (Claparède et Lachman 1858) Penard 1902. (**a**, **b**) original Rothpletz’s illustrations^10^, (**c**) LM photograph and (**d**) SEM image provided by F. Siemensma^42^.

The second oldest fossil diatom record comes from the Middle Jurassic and was described as *Pyxidicula annulata* Rothpletz 1900 (Fig. 6a,b)^10^. *P. annulata* was extracted from a fossilized sponge specimen collected in Bernese Oberland, Switzerland^10^. The location of the type slide containing *P. annulata* and sediment sample used for its extraction is unknown. After determining that the two Lower Jurassic *Pyxidicula* species were not diatoms, we likewise wanted to determine whether similar uncertainties cast doubt on the identity of Middle Jurassic *P. annulata*. We began by carefully analyzing the original publication^10^ to determine the exact locality, sediment type, and sediment age of samples. Rothpletz received the sample from a hobby collector, Mr. Franke-Schmid^10^, who collected the sample “*10 min* *below the Rengglipass* (1800 m) *between the Saxet valley and the Suld valley, on the Suld side in the scree below the Schwalmere* (mountain)’’ (*italics*: direct translation from German page 154). Rothpletz described the sample as a gray limestone plate with dichotomous branches, which he classified as the horn sponge, *Spongelites fellenbergi*^10^. Further evaluation of the sample was done by Mr. E. v. Fellenberg at Rothpletz’s request, as v. Fellenberg reportedly knew the sampling area well^10^. On the geological map revised by v. Fellenberg, the scree was adjacent to a Berriasian outcrop (Lower Cretaceous, 145–140 Ma), but due to the sample lithology, v. Fellenberg made an educated guess that it came from the Middle Jurassic^10^ and arrived at the scree through glacial transport from the Schwalmere peak^10^. However, in the most recent geological map of the area, the Schwalmere peak on its Suld valley side is composed exclusively of Berriasian to Barremian sediments (Lower Cretaceous, 145–125 Ma) with just a small cliff of Malm Limestone Formation (undifferentiated Oxfordian to Berriasian age, 163–139 Ma) at its base^38^. There are no Middle Jurassic sediments in the area, and an Upper Jurassic age of the outcropping Malm Limestone Formation has not been established^38^. Later in 1900, Rothpletz examined additional material collected in Switzerland from the Palfris Formation in the Appenzell Alps, of Lower Cretaceous (Berriasian, 145–140 Ma) age^39^. Due to its morphological characteristics, the sample from Palfris was also identified as the horn sponge, *S. fellenbergii*. This led Rothpletz to conclude that the sample from Bernese Oberland must be of Lower Cretaceous age as well^39^. Although no drawings were provided, Rothpletz claimed to have observed *P. annulata* in the sample from Palfris as well, suggestive of a broad geographic distribution of *P. annulata*, since the sampling localities were 130 km apart^39^. Although we did not carry out sampling in Switzerland specifically for *P. annulata,* during a broad search for Cretaceous and Jurassic diatoms we examined Lower Cretaceous (Valanginian, 140–133 Ma) rock samples from a location in Büls, Switzerland located 7 km from Palfris. No diatoms were recovered from the Büls rock samples.

In conclusion, the information surrounding *P. annulata* and sediments containing these fossils is incomplete and unverifiable. The sample collected from Bernese Oberland comes from scree, located outside of a sedimentary section, and is not a plausible source for extraction of Jurassic diatoms due to its uncertain stratigraphic position. The later sample from Palfris, although reported to have the same origin as the one from Bernese Oberland (a sponge *S. fellenbergii* in marls of the Palfris Formation), also lacks sufficient supporting documentation.

In addition to the issues of reproducibility, the morphological characteristics of *P. annulata* suggest that this species is more likely a testate amoeba, than a diatom. The *P. annulata* fossil in question here is highly similar in morphology to *Pyxidicula operculata* Ehrenberg 1838 and *Pyxidicula patens* (Claparède et Lachmann 1858) Penard 1902, both of which are now considered testate amoebae. In cross-sectional view, *P. annulata* has a thick rim (Fig. 6b) similar to *P. operculata* (Fig. 6c), and the oval shape and punctate surface of *P. annulata* (Fig. 6a) are similar to *P. patens* (Fig. 6d). Rothpletz deliberated on the genus affiliation of *P. annulata* but settled on *Pyxidicula* due to the lack of better taxonomical association and previous use of the name in the Lower Jurassic record, despite the clear morphological differences between those records (the Lower Jurassic specimens from Germany are considerably smaller, have uneven halves with deflected margins, and lack thick rims in the cross-section^9^). Rothpletz focused on the thick inner rim that *P. annulata* exhibits in cross-sectional view, which he saw as similar to the diatom *Galionella distans* Ehrenberg (today *Aulacoseira distans*)^39^. Although *Aulacoseira* exhibits a so-called ringleist in cross-sectional view^40^, which superficially resembles the thick inner rim of *P. annulata*, the ringleist is a unique, apomorphic feature of *Aulacoseira.* The evenly sized halves of *P. annulata* are a known feature of testate amoebae^41^. We conclude that the overall shape and cell morphology, presence of a thickened rim, and densely punctuated surface suggest that *P. annulata* is much more likely a testate amoeba than a diatom. It is properly classified in the genus *Pyxidicula* Ehrenberg. Revisions regarding Lower Jurassic and Middle Jurassic *Pyxidicula* records based on information and developments obtained in this study are compiled in Table 1.

**Table 1:** Compilation table of the most relevant information provided by Rothpletz and developments obtained in this study.

Publication	Rothpletz 1896^9^	Rothpletz 1900^10^
Original species name	*Pixidicula bollensis* Rothpletz; *Pyxidicula liasica* Rothpletz	*Pyxidicula annulata* Rothpletz
Original age estimate	Lower Jurassic (182 Ma)	Middle Jurassic (174–163 Ma)
Sample type and locality	*Phymatoderma* sponge	*Spongiolites fellenbergi* sponge, Switzerland
	Germany	
Original classification	Diatom	Diatom
Main characteristics	Size: 6–14 μm	Size: 30–60 μm
	Morphology: two uneven halves with deflected margins	Morphology: two even halves with straight margins
	Cross-section: circular/elliptical	Cross-section: circular with thick inner rim
	Surface: lattice pattern	Surface: dotted pattern
Revised species name^1^	*P. bollensis* = *P. liasica*	*Pyxidicula annulata*
Revised age^1^	Lower Jurassic ca. 182 Ma	Lower Cretaceous 145–140 Ma
Revised classification^1^	Calcareous nannofossil *incerate sedis Schizosphaerella* Deflandre and Dangeard	Testate amoeba in genus *Pyxidicula* Ehrenberg
Sample type^1^	*Phymatoderma granulata* (trace fossil, not sponge)	*Spongiolites fellenbergii* (undetermined)

^1^Revisions based on results of the present study.

## Discussion

Our search for Mesozoic diatoms demonstrated several challenges in validating the microfossil record and highlighted the importance of extensive and, in some cases, non-traditional examination methods. Although there are examples of fossil diatoms that have been rejected due to age, morphological uncertainties, and taxonomic affiliation, such as reports of Proterozoic diatoms from Poland^43^, the reason for their presence in the sample or what they represent remains unclear^44^. Challenges associated with fossil record validation and interpretation have been encountered in other groups as well. For example, the oldest fossil foraminifera were overturned due to age revision of the sediments containing these fossils^45^. Angiosperms were connected to a broad range of incorrectly classified pollen floras that either represent other plant groups or lack features that might confidently place them within angiosperms^7,46^. Additional precautions in ensuring fossil authenticity such as elemental mapping via SEM–EDS or reviewing the established age of the examined material are not routinely done when describing new diatom fossils and highlight the novelty and robustness of our approach.

We initially identified fossil cysts from the green algal genus *Pleurozonaria* as diatoms, a hypothesis that was overturned when elemental analysis showed that the cell wall was composed principally of carbon, not silica (Fig. 1). Although not commonly used for validation of diatom fossils, elemental analysis proved to be critical for the proper identification of these diatom-resembling forms. Although Rothpletz’s *Pyxidicula* morphologically resembles a diatom, an analysis of the elemental composition might have shown that it, too, was not siliceous, but the methodology did not exist at the time. Our second false positive result was the presence of extant diatom species in the La Voult-sur-Rhône samples, a region that has no nearby adjacent water bodies nor Cretaceous or Cenozoic deposits, demonstrating the ease with which diatoms passively disperse in the environment^47^. Lastly, diatoms extracted from cherts recovered at DSDP site 416A are particularly relevant in the context of *Pyxidicula* species found in the samples from Bernese Oberland, Switzerland^10^. The inability to assess the proper age of samples from an otherwise well-dated DSDP core because of the mixing of cherts between layers during coring demonstrates the importance of verifying a material’s age and potential factors that might have interfered with age determination. The many pitfalls we encountered at these three sites could have also affected the fossils described by Rothpletz, at a time when the methodologies used here were not available.

The evidence presented here challenging the validity of Rothpletz’s *Pyxidicula* fossils is largely circumstantial, so we attempted to reproduce Rothpletz’s collections of Jurassic fossils from Germany. Our *Phymatoderma* samples included calcareous nannofossils of the genus *Schizosphaerella*, a taxon that is not a diatom but whose phylogenetic affiliation is otherwise unknown^36^. The similarities between *Schizosphaerella* and Rothpletz’s two Jurassic *Pyxidicula* species led us to conclude that Rothpletz’s *Pyxidicula* fossils, which he identified as diatoms, were instead calcareous *Schizosphaerella*. Moreover, the two species described by Rothpletz, *P. bollensis and P. liasica*, are very likely the same species, with some specimens exhibiting cell wall deformations due to compaction.

As described above, the third purported diatom fossil described by Rothpletz, *P. annulata*, has all the features of testate amoebae. Reclassification of Rothpletz's Lower Jurassic fossils as the calcareous nannofossil *Schizosphaerella* and Lower Cretaceous fossils as testate amoebae creates broad confusion with regards to the name *Pyxidicula*, i.e., whether it is a diatom, calcareous nannofossil, or testate amoeba. At the time of Rothpletz’s initial publication in 1896^9^, *Pyxidicula* Ehrenberg was considered a spineless version of the diatom *Stephanopyxis* Schütt 1896^48^, which was the primary reason for Rothpletz’s classification of his fossils as diatoms^9^. After the initial description by Ehrenberg in 1838^49^, *Pyxiducla* was considered a diatom and the name was used in the diatom taxonomic literature throughout the nineteenth century^48,50^. However, in 1874 Hertwig and Lesser^51^ revisited Ehrenberg’s record and concluded that Ehrenberg’s *Pyxidicula* was in fact a testate amoeba, not a diatom. Therefore, Schütt’s designation of *Pyxidicula* as spineless version of *Stephanopyxis* was incorrect and came after the reclassification of the genus to a testate amoeba.

*Pyxidicula* remains among the most confusing names in the diatom literature. To fully dissect and clarify the convoluted taxonomical and nomenclatural history of *Pyxidicula*, it appears that (1) inconsistent usage and overall poor documentation, (2) lack of literature citations to (or knowledge of) earlier works, (3) a series of nomenclatural changes involving key species, and (4) changes in our understanding of exactly which phylogenetic lineage “*Pyxidicula*” is thought to represent, have all compounded the uncertainties described above. The use of the same taxonomic name in different groups (i.e., diatoms and testate amoebae) that exhibit similar features (e.g., a cell wall made of two pieces), without original vouchers or type slides, and with descriptions based on drawings, has likely contributed to confusion about the name and identity of Rothpletz’s fossils. Early diatoms in genus *Pyxidicula* described by Ehrenberg and later Pritchard (*P. globota*^50^ and *P. prisca*^52^) exhibit features of testate amoebae, such as a densely punctated surface and thick rim in a cross-sectional view, and should potentially be formally reclassified as testate amoeba. In terms of the taxonomy, Rothpletz’s Lower Jurassic fossils from Germany should be reclassified as *Schizosphaerella*, and the Lower Cretaceous fossils from Switzerland should remain in *Pyxidicula* Ehrenberg, which henceforth should be recognized exclusively as a genus of testate amoebae.

Although predominantly freshwater, testate amoebae have also been recorded from transitional marine-terrestrial habitats from the Late Jurassic^53^. However, Bernese Oberland and Palfris sediments containing the original *P. annulata* described by Rothpletz were deposited on the outer shelf in a fully marine environment far away from any freshwater influence^54^. If the fossils observed by Rothpletz are indeed testate amoebae, their presence in samples could be due to riverine transport to the marine sediments, contamination from a water body in a periglacial environment after the retreat of glaciers, or due to potential laboratory contamination.

*Pyxidicula* (Rothpletz) has been used in many contexts for understanding the early evolution of diatoms, e.g., as a taxon bearing model ancestral morphology^55^. It has been suggested that the appearance of *Pyxidicula* (Rothpletz) may indicate the initiation of silicification in diatoms^16^. It also marks an upper bound for the age of diatoms^5^, so rejecting it creates a 75 Ma gap between the oldest diatom record (Aptian 125–120 Ma, from the eastern Australia deposits^14^) and the inferred crown age of diatoms based on molecular clocks (ca. 200 Ma)^5^. Lower Cretaceous assemblages from Australia are composed of 37 species in 13 genera^14^, and the Weddell Sea assemblage is composed of 47 species in 20 genera^15,16^ of radial and multipolar centric diatoms, indicating that diatoms had been diversifying for a considerable amount of time prior to 125 Ma. However, some important questions remain unanswered: What is the crown age of diatoms? Are they as old as molecular clocks suggest? What is the cause of the 75 Ma gap in the fossil record, and is there any hope of closing it?

The lack of a Jurassic diatom fossil record may owe to several factors: diatoms were not present in the Jurassic, or they might have occupied narrow niches, resulting in a patchy distribution that makes them difficult to find. Early diatoms might also have been lightly silicified, which coupled with low abundance resulted in their absence in the deposited sediment. In addition, most of the oceanic crust from the Jurassic that might have contained sediments with marine diatom microfossils has been subducted, resulting in loss of many of the earliest records^56^. Lastly, the preservation potential of siliceous microfossils may have been influenced by post-depositional silicate diagenesis^57^. Silicate diagenesis is a complex process controlled by many factors such as age, lithology, pH, pore water elemental composition, temperature, and pressure^58^. The Jurassic climate was considerably warmer with no evidence of polar ice caps^59^, hence post-depositional diagenetic pathways may have decreased Jurassic diatom preservation. Taken together, the absence of diatom fossils in the Jurassic is likely an effect of some combination of the aforementioned factors.

Introducing elemental analysis, strict age controls, and the recognition of modern contaminants prevented misinterpretations of otherwise promising fossil discoveries, highlighting numerous difficulties associated with uncovering the deep fossil record of diatoms and other microfossils. The oldest reported diatoms, in the genus *Pyxidicula* Rothpletz, could not be verified, leading to the conclusion that they are most likely not diatoms. Instead, the *Pyxidicula* fossils from Germany are more likely calcareous nannofossils, whereas *Pyxidicula* fossils from Switzerland are more likely testate amoebae. The search for older diatoms should include extensive validation protocols like the ones employed here. Incorporating new fossils into molecular phylogenies may provide a new estimate of the age of the crown group diatoms as well as novel insights into past diversity and the morphological evolution of extinct clades, deepening our understanding of early diatom evolution.

## Materials and methods

### Sample materials

Toarcian black shales were provided by Bas van de Schootbrugge from Utrecht University and recovered by the Schandelah drilling program in 2008^20^. One sample was used in this study.

Lower Jurassic (Callovian) rocks (marls) were collected in La Voulte-sur-Rhône^26^ in France in June 2021. Six samples were used in this study.

Samples from the Posidonia Shale succession (black shales, limestones) (Fig. 5) were collected in Southern Germany in May 2022. Samples were collected across the Posidonia Shale profile in 4 locations: Kromer Quarry in Ohmden^21^ (48° 39′ 8.8122″, 9° 32′ 29.3352″), Aselfingen outcrop^60^ (47° 50′ 38.5938″, 8° 28′ 59.5554″), Dotternhausen site quarry opened for public^37^ (48° 13′ 49.0722″, 8° 46′ 5.829″), and a Bad Boll private outcrop on Reuteweg 6 in Bad Boll (48° 38′ 56.3568″, 9° 36′ 51.8394″). The sample from Bölzhäuser Wald was provided by Guenter Schweigert from the State Museum of Natural History in Stuttgart Germany. In total 19 samples were used in this study.

Cherts and porcellanite of site 416A were recovered by Deep Sea Drilling Project in 1976^28^ and were provided by Bremen Core Repository. We received four samples of Lower Cretaceous age (50-416A-39R-1W, 50–51 cm; 50-416A-39R-1W; 100–101 cm; 50-416A-39R-1, 106–107 cm; 50-416A-39R, 134–145 cm) and one sample of Upper Jurassic age (50-416A-54R-1W, 78–80 cm).

### Chemical digestions

All the samples (except cherts) were cleaned following a combination of common and robust chemical digestions^61^ and personal observations acquired through testing different methods. Approximately 10 g of the sample was crushed to increase the reactive surface and placed in a 300 ml glass beaker. First, 10% hydrochloric acid (HCl) was added and left at room temperature to remove calcium salts. Fresh HCl was replenished daily. After the structure of a sample was destroyed (2 weeks), sediment material was rinsed three times with Milli-Q^®^ water (18.2 MΩ *cm) and treated with nitric acid (35%) (HNO_3_) to remove organic material. Samples remained in HNO_3_ and were replenished daily at 100 °C until the color of the sample significantly brightened indicating removal of the portion of the organic matter (maximum of 4 weeks for black shales). After triple rinsing with Milli-Q^®^, a 33% hydrogen peroxide (H_2_O_2_) solution was added, to continue organic matter oxidation. Samples remained in H_2_O_2_ at a temperature of 100 °C, replacing H_2_O_2_ daily, until the color of the sample was light gray to white (maximum of 6 weeks for black shales). Chemical treatment finished with triple rinsing in Milli-Q^®^. The residual sediments were frozen and freeze-dried in a Hetosicc freezdryer. Dried sediment was then mixed with a sodium polytungstate heavy liquid solution (SPT) of a density of 2.15 g/cm^3^ to extract siliceous parts (diatom frustule density = 2.1 g/cm^3^). The floating fraction was collected in a sterile plastic tube and cleaned from SPT by filtering (0.45 μm cellulose nitrate membrane filter). Collected materials were suspended in Milli-Q^®^ water and used for later examination.

Microscopic slides for light microscope (LM Olympus BX53 petrographic microscope, equipped with a digital camera-Olympus DP28 at the Department of Geology, Lund University, Sweden) evaluation were prepared using 2 mL of the sample solution on a hotplate set to 60 °C. Dried coverslips were glued to the slide with Norland Optical Adhesive 61 (NOA 61) and placed in a UV box for 20 min to allow the adhesive to solidify.

Scanning electron microscope (SEM; A variable pressure Tescan Mira3 High-Resolution Schottky FE-SEM equipped with an Oxford EDS detector at 2 kV was used, housed at the Department of Geology, Lund University, Sweden) samples were prepared from 0.5 mL of the same material. The samples were coated with Platinum–Palladium (Pt–Pd) powder (Cressington sputter coater 108 auto, 20 mA, at 20 s).

Cherts were dissolved using 10% hydrofluoric acid (HF) to dissolve the siliceous structure. The HF protocol provided by S. Gorican^62^ was modified. Before applying the dissolution protocol to the cherts, we tested material containing diatoms of Eocene age to ensure frustule preservation after acid exposure. First, small pieces of crushed chert were placed in a plastic 100 ml beaker and covered with 10% HF solution. After 30 min the acid solution was poured into a plastic filtering container. The remaining pieces of chert were rinsed with Milli-Q^®^ water in the beaker to remove residual acid and dissolved sediment attached to the chert surface. Water used for rinses was also poured into the filtering container. The collected liquid was filtered through a 0.45 μm polycarbonate filter. Sediment collected on the filter was transferred to the sterile 50 ml plastic tube. The remaining piece of chert was submerged in the acid again for further dissolution. All the activities above were repeated after 1 h, 1.5 h, and 2 h. In the end, a set of 5 tubes per sample were obtained: after 0.5 h, 1 h, 1.5 h, 2 h, and a final wash containing the remaining piece of chert. Microscopic slide preparation and SEM sample preparation follow the protocols described above. Note that sediment collected after 0.5 h was not used for LM and SEM examination and was considered a wash run.

### Additional measures

As Callovian marls exhibited the presence of diatoms, additional steps were used to exclude surface contamination. The outer layer of the rocks was removed by a drill and clean fragments of each sample were re-processed in the laboratory following the abovementioned protocol. Raw material from the outer layer of the sample and residue from cleaned rock fragments were reexamined under the light microscope.

Material from the *Phymatoderma granulata* layer was examined unprocessed and following chemical digestion to characterize all microfossils in the sample.

## Supplementary Information

Supplementary Information.

## Data Availability

All data generated or analyzed during this study are included in this published article.
