# Supplementary material for: Uncertainties surrounding the oldest fossil record of diatoms
Source: Sci Rep. 2023 May 17;13:8047. doi: 10.1038/s41598-023-35078-8 (PMC10192206; doi:10.1038/s41598-023-35078-8)
Supplement: Supplementary file 1 — Supplementary Information. [file 41598_2023_35078_MOESM1_ESM.docx]

Supplementary figures


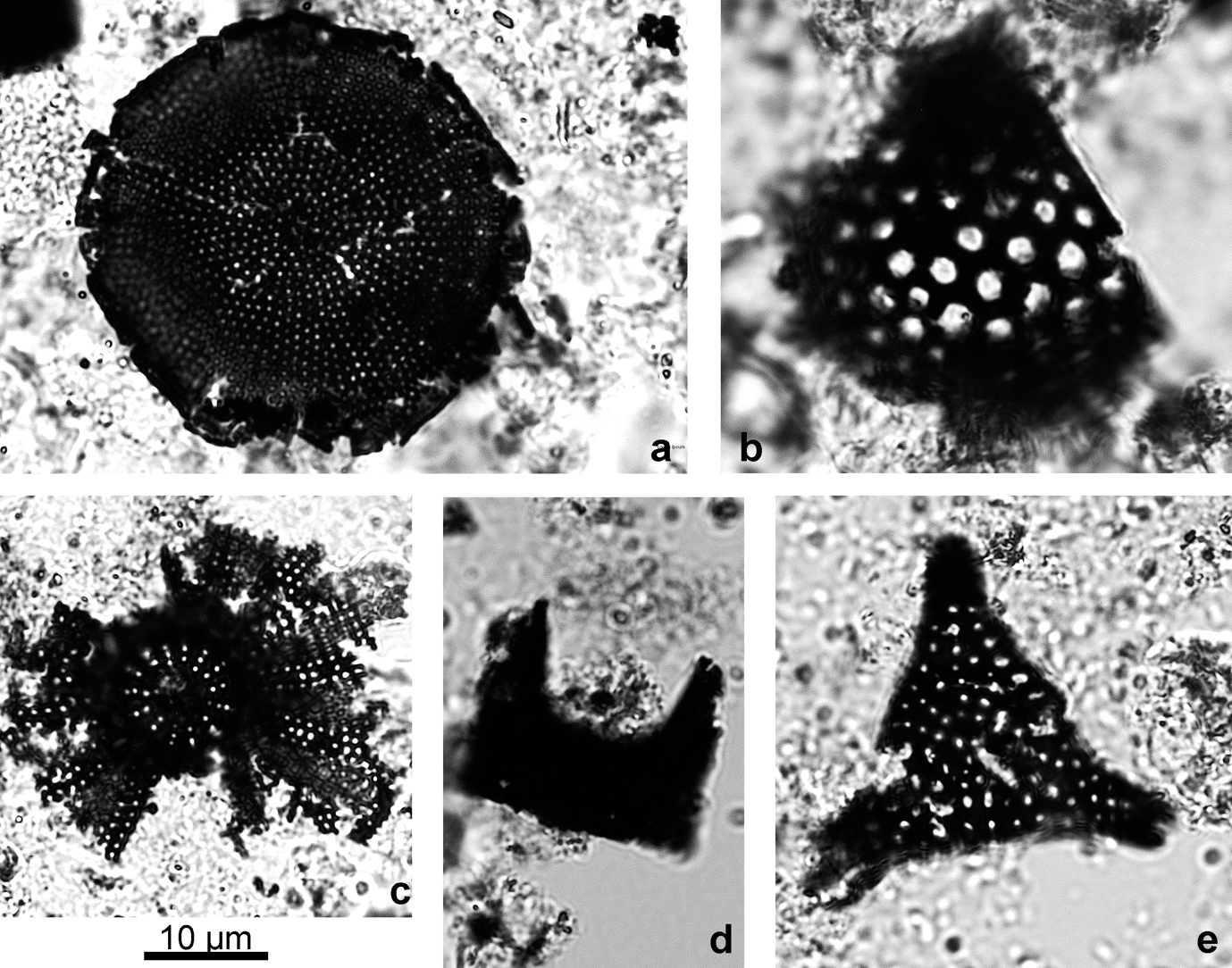


Supplementary Fig. 1. LM photographs of pyritized diatom frustules recovered from cherts of DSDP site 416A^28^*.* Specimens from the sample 50-416-39R-1W, 100–101 cm.


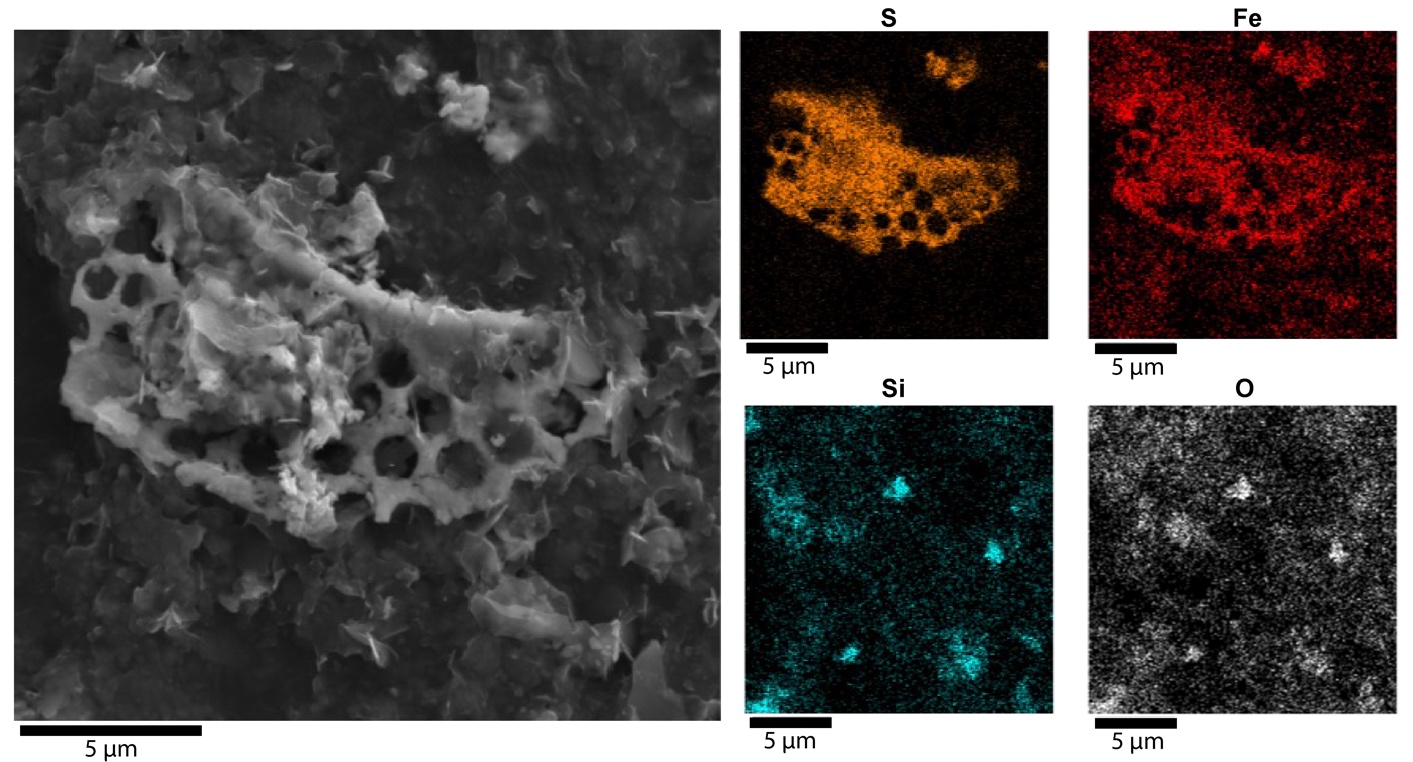


Supplementary Fig. 2. Elemental mapping of sulfur [S], iron [Fe], silica [Si] and oxygen [O] on a diatom fragment recovered from chert of DSDP site 416A^28^. Specimen from the sample 50-416A-39R-1W, 100-101 cm.


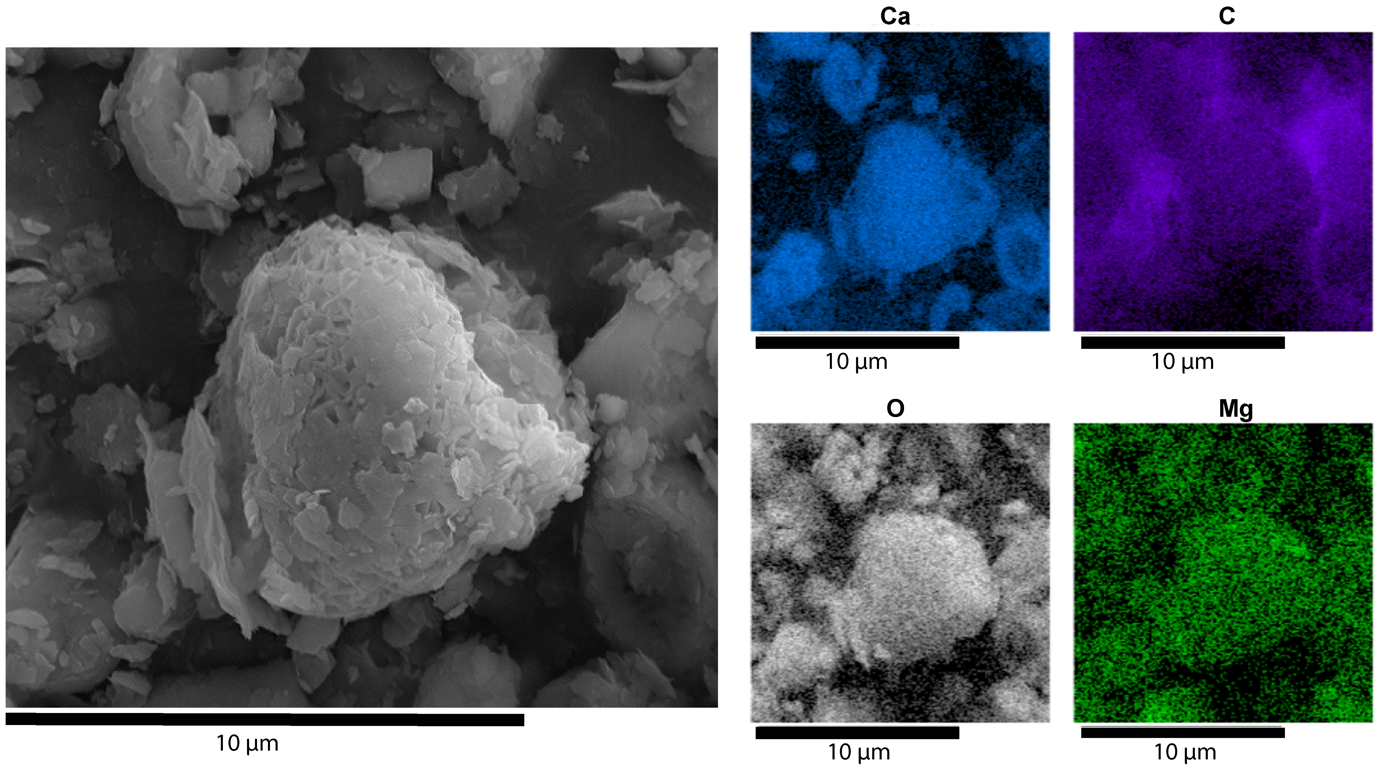


Supplementary Fig. 3. Elemental mapping of calcite [Ca], carbon [C], oxygen [O] and magnesium [Mg] on *Schizosphaerella* specimen recovered from *Phymatoderma* sample.
